# Thirty-Fifth Annual Meeting of the Michigan State Dental Association

**Published:** 1890-07

**Authors:** 


### THE DENTAL REGISTER.

VOL. XLIV.] JULY, 1890. [No. 7.

### PROCEEDINGS AND PAPERS

### Of the Thirty-Fifth Annual Meeting of the Michigan State Dental Association.

The first session was called to order at 10:30 A.M., June 3d, in the council room of the city of Jackson by C. S. Case, the president. Rev. Geo. S. Hickey offered prayer.

Edwy Knight, Esq., then addressed the meeting, welcoming the dentists to the city and thanking the association for the honor conferred on the citizens of Jackson not only in selecting this city as the place of meeting, but also in choosing one of their number as president. He expressed the opinion that it was not only the central location of the city, but the recognized hospitality of her citizens that determined the choice of the place of meeting this year at Jackson. He then extended the hospitalities of the city, its public and private institutions as well, to the members of the Association.

President Case thanked the mayor for his generous welcome in a few well chosen words. The president announced the death of Dr. C. H. Dyer, of Grand Rapids, and stated that by his death the Association was left without a first vice-president. On motion Dr. E. C. Moore, of Detroit, was elected to fill the unexpired term of Dr. Dyer.

A letter was read from Dr. Louis Ottofy, of Chicago, asking that the secretary of the Association be instructed to send to him a report of any matters of interest that may be brought before the association, to be referred to the chairmen of the different sections of the American Dental Association, to be reported at the next meeting. On motion this letter was referred to a special committee to investigate and report upon. Morning session adjourned.

The afternoon was given up to clinics. Dr. W. A. Dorland, of Grand Rapids, made a gold filling involving the mesial approximal side and part of the labial surface of a superior lateral incisor. Pellets of gold rolled from No. 4 foil were used, and the Snow & Lewis automatic mallet was used to pack the gold. The filling was a success and demonstrated not only the skill of the operator but the rapidity and thoroughness of this method of using foil and the manner of packing it when skillfully done.

Dr. J. L. Gish, of Jackson, showed a Rheostat of his own invention. The instrument is designed to take the electric current from the street wire used in electric lighting, and so modify it as to make it applicable for mechanical and therapeutical uses in the dental office. By means of this apparatus the current can be brought from the slightest manifestation to the full capacity of the street wire in a perfectly increased current or power without the intermediate shocks of other such devices caused by jumping the switch from one post to another. This feature makes the apparatus invaluable for therapeutical purposes, as it renders the application of electricity to the living tissue almost painless and at the same time sufficient power can be secured for the accomplishment of any desired effect. In fact, this appliance puts a most valuable agent at the service of the dentists.

Dr. C. S. Case demonstrated his method of making the "angle system" of regulating appliances and illustrated several cases of practical application. The doctor used German silver plate and wire and illustrated the method used in making the tubing and screws. A piece of plate is cut of sufficient width to make the required size tubing when the edges have been turned up together. This is accomplished by drawing it through a wire draw-plate, beginning in the larger holes and passing it successively through the smaller ones until the proper size is reached. The tubing may be soldered or not as the case may require. The doctor, instead of using a vise to hold his draw-plate, had two iron straps bent to the triangular shape and fastened to his work bench with strong screws. The draw-plate is placed inside of these irons and held against the base by a wooden pin. The advantage of this device being that the top of the plate is held firmly in place as well as the bottom and free access is obtained for the draw-tongs.

The screws are made with an ordinary screw plate; the nuts are cut from five cent nickels. A variety of appliances were shown which can not be described intelligently without drawings. But a great variety of independent or concerted movements may be had by this form of apparatus. Every case will suggest some variety of the pull or push movement that can be easily accomplished by some variety of this system. The teeth are banded in almost every case so that there is no mutilation of the teeth. The apparatus is easily made by any dentist of mechanical skill. The results secured by Dr. Case and the great saving of time commend the system to all who value time and the comfort of their patients.

EVENING SESSION.

Meeting called to order at 8:30 P.M. Minutes of previous meeting read and approved. Dr. C. S. Case of Jackson, the president of the Association, read the following :

### President's Address,

Delivered before the Thirty-fifth Annual Meeting of the Michigan Dental Association, at Jackson, Mich., June 3d, 1890,

BY C. S. CASE, M. D., D. D. S.

At this, the thirty-fifth, annual meeting of the Michigan Dental Association I again have the honor of addressing this body as its President, and while I can but justly feel proud of the distinction of a second term as your presiding officer I regret that your choice had not fallen upon some one of the many whose executive ability would have enabled them to serve you in a more able manner. But I am consoled by the thought that you will accord to me your usual aid and indulgence should questions arise that require more than ordinary knowledge of parliamentary rules.

I think I may say with some little pride that the meeting a year ago at Grand Rapids was an exceptionally good one in every particular; and now if this meeting proves as successful as we have every reason to expect—judging from the program and the many assurances of a large attendance—it may be taken as *prima facia* evidence (and we congratulate ourselves upon the fact) that the Michigan Dental Association has arrived at that stage of development and maturity which makes it no longer necessary—as was believed in former years—that our meetings must be held in Ann Arbor, Detroit or any other special locality in order to assure that success which will make them worthy to be called the annual meetings of a State dental society. In fact, I am still of the opinion, as expressed in my address of a year ago, that western and northern portions of the State should cease to be shunned in selecting places for our meetings. There is a large number of dentists in these localities who rarely if ever attend our conventions or take the slightest interest in their proceedings, but who, I feel assured, would become more prominent, and an honor to the profession if some inducement could be thrown around them to enter our fold, with its influences for professional attainments and conduct, and the only way to accomplish this result is to hold our meetings occasionally in their vicinity.

We have a number of live cities west and north from which to make a choice. Among these may be mentioned Kalamazoo, Battle Creek, Niles, Saginaw and Bay City. Nor should we forget that our State affords many delightful summer resorts, where, if it please the Association, we could combine the pleasures and rest of an outing with the profit and instruction of a dental meeting.

No need to have the slightest fear of failure wherever you decide to go if you are careful in the selection of your officers. Choose men who are not only able but willing to do the work assigned to them, men who will use their best efforts to fill your programs so full of interesting and instructive things which pertain to dentistry proper that there will be no more room for those lengthy and tiresome displays which have characterized some of our meetings in the past—things which may be interesting and desired by a few, but as I understand it, not the purpose or aim of this society. The majority of us are here to learn something practical about dentistry, and in order to make these meetings rousingly successful we must always be able to go from them with the feeling that we have been benefitted and are more capable of fulfilling the demands of our profession.

The dental society should be looked upon as a school, or if you please a practitioners' course, organized and run for the sole purpose of giving information to its members in regard to the most advanced thought and methods of a fast progressing profession. Here we should expect to find men who are vastly our superiors in special branches, because they are men who have devoted the main portion of their professional thought and energy to special channels into which they have naturally drifted by peculiarities of ability, and by whom we should be as glad to be taught as a student by a professor in a dental college.

There is an unfortunate characteristic of this Association that I have not noticed displayed, to such a marked degree at least, by other dental societies. Some of our members who stand in the front rank of the profession, who have gained a high reputation both at home and abroad, are the last ones to willingly contribute papers or clinics to our program. When they are asked, we are told they haven't time, etc., and yet we often see their names as authors of interesting articles and papers read at other societies. It would almost seem as if they considered it rather beneath the dignity of their high position to read a paper or show their manipulative skill and methods before this little Michigan Association and that it was sufficient if they honored us with their presence and critically discussed the arduous efforts of the executive committee to make these meetings interesting and instructive.

We were told by some of these gentlemen at our last meeting that clinics at dental society meetings were as a general thing a failure; that one reason why the best operators would not operate was because the facilities afforded would not admit of their doing the work as perfectly as in their own offices, and many other things were said calculated to dampen the ardor of those who had worked so hard to make this modern and admittedly instructive branch of advanced dental societies a prominent feature of *our* Association. It is not expected, I think, that one can produce as perfect an operation at a dental clinic as when surrounded with the conveniences and unobstructed facilities of one's office. But it is not the final result of an operation that gives instructions. It is the *methods* persued by the operator in his efforts to produce a certain result. The instruments and material that he uses and the manner of manipulating them. It is not supposed that men will come here to perform experiments or any operation which they have not repeatedly and successfully performed elsewhere, nor one so odd that the result in any event would be a matter of question; therefore, whether the operation is as perfectly performed here as it would have been under other circumstances is a matter of secondary importance so long as we have seen the ways and methods of a skillful operator. It is not claimed that it would be particularly instructive at this day to see the rubber dam applied or gold foil packed into a cavity, however graceful and rapid the operator or perfectly contoured and polished the filling unless there was some unknown and helpful method or material used. But I believe that a large proportion of the men who attend these conventions would be benefitted by seeing a skillful dentist *approach* a dead or exposed pulp in the most approved manner; his method of treating, capping or filling root-canals; the preparation of a cervical border; the use and application of new and approved matrices; the method of obtaining space for properly contouring a filling; the exhibition of new and useful preparations of gold and the most approved manner of manipulating them, and many other things. Then if that operator would consider it a part of his business to use his tongue as well as his hands many of us would go back to our offices with new knowledge, thoughts and determinations impressed upon us with a force which no amount of study or didactic method of instruction could begin to equal.

If this view of the question was more fully appreciated and entertained by those members of this Association whose skill has gained for them a name in whatever land the profession of dentistry is practiced then our clinics might become not only unquestionably instructive but by far the most attractive part of our meetings.

It is not too much to say that large numbers who rarely attend our meetings would be on hand if Drs. Taft. Watling, Field and other notable operators were down for a clinic, and how many of *us*, think you, would not be greatly benefitted by such an exhibition?

A few days ago I stood by the chair of one of the above named gentlemen for a few minutes and while urging him in my most persuasive tones to do something for us at this meeting I caught an imperfect view of a narrow yellow ribbon, which looked like Williams' crystalloid or electric gold, being folded rapidly back and forth into a cavity and condensed with the electric mallet. As I turned away I could but realize—as I believe to-day—that if that simple operation, which was so common to him, could have been seen by many a graduate and even older practitioner who had been taught only the use of foils and other methods, its instructive influence would have been incalculable.

In the fall of '70, after being with Dr. Jerry Robinson for nearly five years as student and partner, I had the pleasure of seeing Dr. J. Taft put in a gold filling at Cincinnati. Up to that time I had seen but one man operate and this opportunity was simply a revelation to me. At that time the rubber dam was in its infancy and the loud blowing whirr of a pneumatic dental engine—a primitive form of this machine—was among the wonders that so impressed that whole operation upon my mind that I could now describe its minutest detail. And even had I received no further instruction from that hand, the opportunity of seeing that simple operation would have been a great benefit to me. I don't mean by that that the work was especially remarkable or superior to that which I had seen before, but it showed me a different way;—a thing that is as possible among dentists of to-day as then. One reason why men cling to old, and sometimes imperfect methods, is because there are no opportunities offered them for *seeing* the ways of others.

A skillful dentist, of over twenty-five years' experience, said to me the other day that he had often seen the time that he would have gladly given ten dollars to be allowed the opportunity of going to the offices of other dentists in his city and watch them operate. This was not an indication of weakness or inability but rather the healthy and natural desire of an experienced operator who had developed to that degree which enabled him to recognize the special ability of others; and what is more, it was a yearning for that method of instruction which can not be obtained from papers, periodicals or text-books.

Excepting young graduates, whose high estimation of their own ability is always to be expected, the class of dentists in every community who imagine they can be taught nothing are usually the ones who are the most imperfectly informed. They never seek knowledge of their local confreres, whose capabilities they are ever ready to underrate and whose methods they believe far inferior to their own; nor do they devote much of their time to reading the current dental literature for fear, I suppose, it will interfere with the transcendentalism of their own thoughts and methods, and they rarely attend dental conventions, whose proceedings they not unwillingly slur as unimportant.

These remarks do not apply, however, to all who do not join dental associations, for I am acquainted with a number of dentists in this category who possess superior qualities and ability.

A few months ago I saw two large approximal cavities in inferior bicuspids filled for a young but skillful dentist whose business push had given him considerable notoriety and a large practice, and who, I have reason to believe, imagined he was doing work according to the most advanced and approved methods. There was used in this operation a Freeman matrix, Perry separator, Williams' crystalloid and rolled platina and gold, the whole operation being done with an ease and rapidity that was something of a surprise, I imagine, to the patient. Up to that time he had known nothing of the working qualities of the Perry separator or crystalloid gold. Here he was forced into a peculiarly favorable position for judging by observation and experience of the usefulness of two modern radical improvements in dentistry, which, I am pleased to say, produced such a favorable impression that he immediately sent for a whole set of the separators and commenced using the crystalloid gold. And yet had this operator, who with considerable reason prides himself upon being at the top, visited a number of important society clinics he might have seen the practical application and advantage of these implements at a much earlier date,—to my certain knowledge as early as the meeting of the medical congress at Washington.

At a clinic of the American Dental Association I saw a matrix adjusted by Dr. Darby for a filling in the post-approximal surface of a superior bicuspid, the cavity being filled with remarkable rapidity by a New York City dentist by the Herbst method with Walrab gold. When the matrix was removed the palatine border of the filling was found to be quite imperfect, and consequently, so far as the filling was concerned, the exhibition was a decided failure. And yet that whole operation—the exhibition of peculiar implements, material and methods—was fully as instructive as it could have been had the result been as perfect as it no doubt would have been in the hands of an Herbst or under more favorable surroundings. And while this slight and most natural defect had its lesson, the clinic on the whole was a success and did not deter me from employing the method, as I have done many times since in a certain class of operations, with the greatest satisfaction.

From these few examples which have fallen under my own observation I think we may draw a lesson in favor of making clinics and practical demonstrations a more prominent feature of our association, and of ridding ourselves of the fantastic notion that one is *expected* to operate here with the same graceful ease and perfect skill that he would at home, or that it is the final result and not the whole operation itself that should be considered the instructive part. And further, even the best of us, if debarred from seeing the application of new and improved methods which others more ingenious adopt or invent, are liable to fall into a rut, and, too, while imagining that we have arrived at the acme of professional ability.

There is another side to it not less important to the members of this Association who hope for a larger attendance and the full development of that latent ability, especially among the younger members of the profession in this State. Many men will not afford the time and expense of attending a dental meeting which offers no attractions for knowledge other than will subsequently appear in the published proceedings. Nor can we hardly expect men to come here for the sole privilege of *seeing* dentists say that which can be leisurely read and more perfectly digested at home without the expenditure of time and money, to say nothing of the necessity of sitting for hours listening to the display of red tape and much that is as tiresome as it is uninstructive. If we can not put up something here, a complete knowledge of which is unattainable at home, something which no published report can adequately describe, something which must be seen in order to be fully appreciated, then these dental meetings —outside the social attractions which they offer, the interchange of personal ideas and experiences—*are*, in my humble estimation, a failure.

In regard to papers. It is quite surprising to find so many able men unwilling to write a short resume, to be read at a dental convention, of some one of the many experiences of practice which have a practical and instructive bearing. It isn't possible they have no original thoughts worth listening to, that their experiences never get out of a routine rut, or that they fear to say on paper exactly what they are doing because of a just criticism which it may provoke. It may be partly due to the belief that a paper in order to be valuable to a convention of dentists must always be relative to something new and peculiar and possess certain stereotyped properties and proportions which would necessitate the expenditure of considerable study and thought. While this is true of many subjects which pertain to dentistry, there are others equally important upon which many of us are more or less rusty or far behind the times and possess so little scientific knowledge that there is always room for instructive essays from those who are well up in the most advanced principles, even if they contain no more than a description of the common methods of every day experience.

When I asked a prominent dentist to write a short paper on the treatment of a certain stage of alveolar abscess he at first replied by asking if I didn't think "that subject a good deal of a chestnut?" So long as professional men differ widely in their treatment of a common pathological condition; so long as carbolic acid and a free admixture of saliva continues to hold undisputed sway as the *ne plus ultra* in the offices of many experienced dentists, while other and equally, if not more, important drugs are silently tabooed or openly denounced as unworthy a place in our dental materia medica, the subject is most certainly not a chestnut; or if it is, it is one that should continue to be opened on every occasion until there is a more common acceptance—or at least a more general understanding—of the methods and medicaments used by dentists of acknowledged skill and which are to-day advocated by the most advanced thought.

If I could say anything to induce the *live* dentists of Michigan to spill a little more ink for the benefit of their brothers and this Association I should feel repaid for any effort. But I will not ask you, as did a recent president of a dental society of its members, to commence now to prepare your papers for our next meeting a year hence, for I should expect that the subject, unless a peculiar one, would become stale and worn out by much handling. I would, however, most respectfully suggest that it be remembered by great and small that the principal purpose of these yearly meetings is to examine and discuss thoughts and methods which are new and worthy to be applied practically to our professional work. All that we ask is the idea itself, presented in concise English, and if it covers no more than half a page of foolscap so much the better. We will manage to exist if it doesn't contain all that has or may be written upon the subject, or if it comes to us unclothed by eloquence and flights of oratory. I believe there is no one within sound of my voice incapable of presenting an original thought or idea sufficiently intelligible for practical purposes if he will but hold himself strictly to the subject in hand. The great fault with men, and especially with beginners who contemplate writing a paper, is they imagine it must be constructed on architectural principles, which immediately loom into such proportions of structure, detail and finish, the common result is—no paper at all, and so "many a gem," etc. If every member would do his best toward furnishing something instructive to this association at its future meetings, either in papers, clinics, or demonstrations, our sessions would not only require to be extended but the Board of Supervisors would need to transact much of the business of the Association—as suggested in my address of a year ago—that valuable time which is now wasted to members in convention could be wholly used in the examination of subjects which pertain to the practice of dentistry.

There are many members who pride themselves upon their regular attendance at our meetings and feel they are doing their whole duty, yet with no attempt or expectation of doing anything but to absorb and be benefitted by the hard work and accomplishments of others, giving nothing in return, not even the expression of a single idea in discussion. Now I have great sympathy for those who can not speak in public, and I am aware also that in nearly every convention there are some that express themselves in smooth and flowing language, who are known to be far inferior in manipulative skill and the practical application of ideas than others who say nothing, but this is no excuse for the latter class refusing to do something in other departments.

There is another subject to which I wish to direct attention. Our constitution tells us that the object of this Association is "to elevate and sustain the professional character of dentists, and to promote among them mutual improvement." I believe this to be not fully appreciated or taken cognizance of in its broadest and most useful sense. In our endeavors, as an Association, to sustain and elevate our own professional status, it should be considered equally important (according to my interpretation) to strive for the elevation and improvement of those who are at present without the pale of this society and its influence, and who in consequence are pursuing practices that are not only in discord with professional ethics but far inferior to that which they might attain to were their field of opportunities allowed to be enlarged.

I am aware that I am treading upon an old battle ground and have no desire to raise a point that will cause us a useless expenditure of time in debate. I think all will agree that the true and manly way to help a man who is wallowing in the mire of ignorance or unprofessional acts is not to scoff at him from some far off pinnacle, as one unworthy to be associated with men of professional attainments and character but to kindly extend a helping hand, endeavor to surround him with influences which will tend to educate and perfect him, and, what is more than all the rest, strive to make him feel the responsibility of maintaining a position of honor which has been intrusted to his keeping. If such men are made to feel that they are forever ostracised from membership in this society and looked down upon by its members because of a lack of ability or for pursuing methods which they believe to be for their pecuniary interests, then nothing better can ever be expected of them.

I respect the man who, from lack of skill or business ability, boldly advertises to do work for half price if need be to give proper support to himself and family. And even though in doing this he is shut out from the benefits of this Association, that man is a king compared to the member who, in possession of social and professional position and education, adroitly improves every opportunity offered by the press and otherwise to lay claim to a position in the profession which he does not possess, and for the sole purpose of falsely elevating himself in the minds of the community where he has chosen to practice.

But if men have by their work and skill, risen to the honor of being chosen by national, international or other important dental association to read a paper or perform a clinic I can see no objection to allowing a modest mention of it to be made in their locality; but anything further than this—such as using the trust in a systematic course of advertising, or in an attempt to represent that their special operation (which in reality may have been an exhibition of no special skill) was one of the wonders of the convention—is, to say the least, an exhibition of weakness, a lack of a proper sense of professional and "eternal fitness of things" and one which will more often redound to their discredit in the community, to say nothing of the inevitable lowering of their social and professional standing among their confreres.

There are many other things which I as your president believe it my duty to bring before you, but I fear I have already tired you with this somewhat lengthy address. I hope I have said nothing which will be taken other than in the kindly spirit intended; and if at times I have seemed severe it is because I have the future welfare of this Association and the general elevation of the profession of dentistry in Michigan at heart.

In leaving the chair which I have had the honor to fill for two years and three months to a more able man, the knowledge that I and my efficient corps of helpers have succeeded in making the meetings of '89 and '90 successful and satisfactory to you will be one of the proudest moments of my life. The honor, if any, belongs to those who have done so much to aid me and to whom I now extend my most heartfelt thanks.

One has gone from among us to return no more forever whose aid at our last meeting in providing for our social entertainment and pleasure will long be remembered with most grateful feelings. Dr. C. H. Dyer, of Grand Rapids, First Vice-President of this association, died at Mt. Clemens Springs, December 27, 1889. His death was a sudden and unexpected blow to his family and host of friends who remain to mourn the loss of a kind and loving husband and father, a true friend and genial companion. To this society the loss is a peculiarly sad and unfortunate one, for he combined qualities which would have been of the greatest help to us at this and our future meetings. To myself it is especially sad, for in a brief acquaintance I had formed a high regard for his personal qualities and counted much upon his executive ability to aid me in the proper discharge of my duties at this meeting. I would suggest, in closing, that a committee be appointed to draft proper resolutions expressive of the feelings of this Association.

---

On motion the president appointed Drs. Dorrance, Owen and Wood a committee to prepare suitable resolutions on the death of Dr. C. H. Dyer.

Dr. Geo. L. Field, of Detroit, thought the address of the president the best he had ever heard on such an occasion. He could most heartily sustain him in every position he had taken. He moved that a vote of thanks be extended the president for the able address he had given at this meeting. The vote was unanimously carried.

Dr. W. H. Dorrance, of Ann Arbor, wished to call attention to one feature of the address, which he wished particularly to emphasize, and that was, that too much time was taken up in meetings discussing matters of business and especially when the occasion chanced to be the disciplining of some member for disregarding the code of ethics. Almost every meeting is largely taken up with the consideration of some such business to the exclusion of valuable discussions on scientific subjects of vital interest to the members of the Association. He suggested a change of the constitution that would provide a committee to whom all such subjects should be referred, if not for final action, at least for preliminary consideration and recommendation.

Dr. J. A. Swasey, of Chicago, thought the address was full of grand suggestions. The reference in the address to the fact that our best men were not willing to appear as clinicians at dental society meetings, reminded him of the great benefit he once derived from a visit to the office of Dr. J. H. McKellops, of St. Louis. He described the manner of reception given him, and how he was invited to come into the operating room where the doctor was working for a very refined and well dressed lady, to whom he was introduced, and, upon request of the doctor, he was graciously allowed to remain and witness the operation of filling a tooth, receiving great instruction from the methods and manner of using them, with the comments of the doctor. He thought more clinics of this kind would be beneficial at society meetings especially by men of high attainments.

Dr. Rix said he agreed with Dr. Swasey, but he did not know how we were to have the advantages of these office visits, for it seemed to him almost impracticable to gain admission to the offices of these great men. He had some times done so, but he felt that one was compelled in a large measure to suppress all feelings of modesty and call upon his reserve assurance in order to gain admission to the operating room of men from whom much could be learned. If these men will not give public clinics or admit you to their operating rooms, how can we expect to get anything from them?

Dr. Douglas referred to the time, forty years ago, when it was altogether impossible to gain admission to the operating room or even the laboratory of a dentist. If we complain of these things in this day, how much more unfortunate were we then when there were but few dental journals and societies, and only a few who were willing to describe their methods to a brother practitioner.

Dr. Metcalf, of Kalamazoo, being called out by Dr. Field, said he could not attack any position taken by the president, as he was thoroughly in accord with all he had said, and he could but emphasize all that had been said, therefore there was no chance for an argument for him, but he believed there were men in the house that had been hit by the address and he would call upon Dr. Field as one of them to get up and defend himself.

Dr. Field, of Detroit, thought the address was full of meat, no skin and no chestnut; agreed most heartily with all the positions taken by the address. I want to call attention to one feature of the address in particular, and that is to the practice of a man occupying an honorable position or standing in the profession, of making use of questionable means to keep his name prominently before the public. It is surprising to see the methods that are made use of to notify the general public that a certain distinguished or well known dentist of this or that town has been highly honored in some unusual way. It usually appears in the local paper, and of course the irrepressible newspaper reporter is to blame for it. But all such tricks are unworthy of a gentleman and lowering to the dignity of the profession, if not an actual violation of the code of ethics.

Dr. W. H. Jackson, of Ann Arbor. We talk too much about violations of the code of ethics. What we should do, would be to take notice of these offences and hunt them down, establish the responsibility and place it where it belongs; then prefer charges and have the offenders punished as they deserve.

I would like to emphasize what has been said in regard to witnessing the operations of such men as Drs. Field, Metcalf, Hawxhurst, Allport, Swasey, Crouse and others. It was a great benefit to me to be permitted to witness these men in their own operating rooms.

Dr. Harroun, of Toledo, criticised the manner of conducting clinics. There is too much crowding around the chair of the operator, too much interrupting of the operator by useless questions. Some persons imagine they are the only ones to receive any benefit and so monopolize the entire space about the chair as well as the operator's time. The operator should provide himself with his accustomed surroundings as nearly as practicable, and explain his methods in a systematic way, illustrating them as the work progresses. It is the method we are particularly interested in, not so much the wonderful skill of the operator.

Dr. Case thanked the association for the hearty endorsement given his address, and declared the next order of business to be the reading of a paper by Dr. L. D. Wood, of Grand Rapids, entitled,

